# The Role of Notch Signaling in Macrophages during Inflammation and Infection: Implication in Rheumatoid Arthritis?

**DOI:** 10.3390/cells9010111

**Published:** 2020-01-02

**Authors:** Esra’a Keewan, Saleh A. Naser

**Affiliations:** Division of Molecular Microbiology, Burnett School of Biomedical Sciences, College of Medicine, University of Central Florida, Orlando, FL 32816, USA; Esraakeewan@knights.ucf.edu

**Keywords:** rheumatoid arthritis, notch, macrophages, cell signaling, inflammation, toll-like receptors, RBP-J

## Abstract

Notch signaling coordinates numerous cellular processes and has been implicated in many pathological conditions, including rheumatoid arthritis (RA). Although the role of Notch signaling in development, maturation, differentiation, and activation of lymphocytes has been comprehensively reported, less is known about its role in myeloid cells. Certainly, limited data are available about the role of Notch signaling in macrophages during inflammation and infection. In this review, we discuss the recent advances pertaining to the role of Notch signaling in differentiation, activation, and metabolism of macrophages during inflammation and infection. We also highlight the reciprocal interplay between Notch signaling and other signaling pathways in macrophages under different inflammatory and infectious conditions including pathogenesis of RA. Finally, we discuss approaches that could consider Notch signaling as a potential therapeutic target against infection- and inflammation-driven diseases.

## 1. Introduction

### 1.1. Notch Signaling and Its Core Components

Notch signaling is a juxtacrine signaling that mediates cell-to-cell communication through the receptor–ligand interaction between neighboring cells [1]. It is a highly conserved regulatory pathway that is found in all multicellular organisms from *Drosophila* to mammals [1,2]. It coordinates a vast array of cellular processes including cell proliferation, metabolism, differentiation, and cell survival during development and adult life [3,4]. In mammals, there are four Notch receptors including Notch 1-4 and five ligands including Delta-like (DLL) 1, 3, and 4 and Jagged 1 and 2 [1]. The receptors are based on a single-pass type I transmembrane protein, which is synthesized as a single precursor protein before it undergoes proteolytic cleavage by furin-like convertase at site S1 in the Golgi complex, producing a non-covalent associated heterodimer protein at the cell surface [3,5]. Each Notch receptor is composed of two functional domains including the Notch extracellular domain (NECD) and the Notch intracellular domain (NICD) [2,5]. Classically, NECD consists of 29–36 epidermal growth factor (EGF) motifs that mediate the ligand–receptor interaction, followed by Lin-12-Notch repeats (LNR) that avert unspecific receptor activation. NICD has a transcriptional activity and contains several functional elements including a PEST (proline/glutamic acid/serine/threonine) domain, ankyrin domains, recombinant recognition sequence binding protein at the J Kappa site (RBP-J)-association module (RAM) domain, and nuclear localization signals [3,4].

### 1.2. Intracellular Notch Signaling Cascade

Mechanistically, the canonical Notch signaling is induced by the physical interaction of Notch ligands and NECD of neighboring cells. This ligation triggers consecutive cleavage events as follows: the first cleavage is extracellular cleavage at site S2, and it is mediated by a disintegrin and metalloprotease (ADAM 10 and 17) to shed NECD, which eventually endocytosed by the ligand expressing cell. This is followed by cytoplasmic cleavage of NICD at site S3 by the γ-secretase complex [5,6]. This is a rate-limiting step of Notch signal activation, which could be pharmacologically inhibited by γ-secretase inhibitors [7]. Once NICD is released, it translocates to the nucleus, where the RAM domain interacts with DNA binding protein CSL (C promotor-binding factor 1 (CBF1)/RBP-J in humans, suppressor of hairless in *Drosophila melanogaster*, Lin-12 and Glp-1 (Lag-1) in *Caenorhabditis elegans*), which leads to converting CSL to a transcription activator through displacing of co-repressor complex and recruits transcription co-activator (Co-As) such as mastermind proteins (MAML 1-3) and CREB-binding protein (CBP)/p300 to transcriptionally activate Notch target genes. In the absence of NICD, CSL acts as a sequence-specific repressor to the promoter area of target genes by recruiting transcription co-repressors (Co-Rs) and histone deacetylases (HDACs).

**Figure 1:**
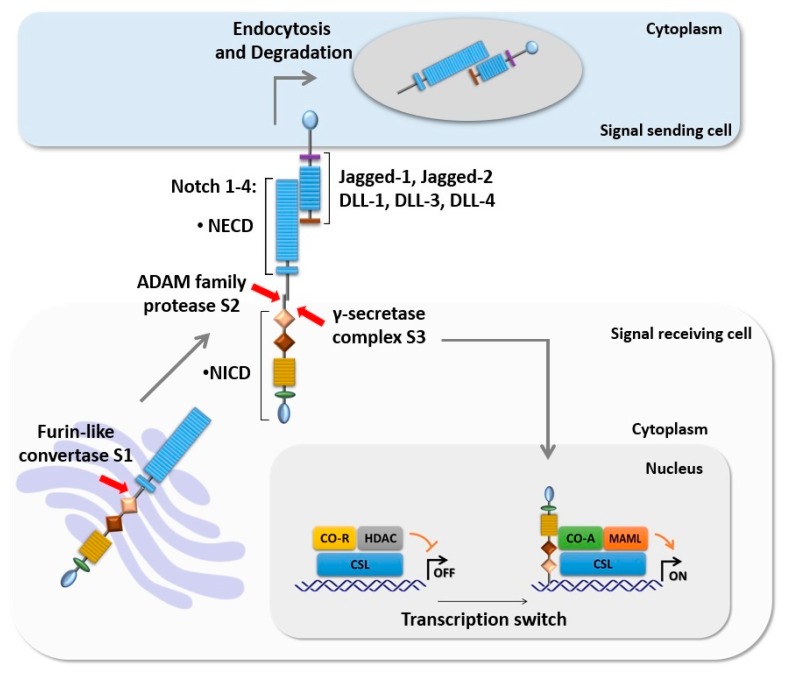
Intracellular Notch signaling cascade. The core components of Notch signaling are Notch ligands (DLL-1, 3, and 4 and Jagged-1 and 2), Notch receptors (Notch 1–4), and the CSL transcription factor. The newly synthesized Notch receptor is processed in the Golgi apparatus by furin-like convertase at S1. Activation of Notch by its ligand initiates two proteolytic cleavages of Notch at S2 and S3 respectively. S3 cleavage results in translocation of NICD to the nucleus where it mediates transcription switch through displaces of CSL CO-R and recruits CSL CO-A such as MAML, promoting the transcription of the target genes.

In mammals, a significant number of Notch target genes have been recognized recently in various cell types. The most common Notch target genes are members of the basic-helix-loop-helix transcriptions factors belonging to the hairy and enhancer of split (HES) and HES with YRPW motif (HEY) families [4,5,6] (Figure 1).

While the canonical Notch signaling cascade involves the ligand–receptor interaction, receptor cleavage, and CSL transcriptional regulation, the non-canonical Notch signaling might be activated by a non-canonical ligation or in the absence of Notch receptor cleavage, and in some cases independent of CSL interaction [6]. The non-canonical Notch signaling induces expression of distinct genes through crosstalk with other signaling pathways including nuclear factor-κB (NF-κB), wingless-type MMTV integration site (Wnt), transforming growth factor-β (TGFβ), Toll-like receptors (TLRs), and hypoxia-induced signaling pathways [6,8].

### 1.3. Notch Signaling in Inflammation

Intracellular Notch signaling regulates numerous cellular processes during development and adult life [3]. Thus, it is not surprising that dysregulation in Notch signaling pathway has been implicated in several pathological processes including inflammatory diseases [9,10,11,12,13]. Most of the data regarding the role of Notch signaling in inflammation are based on manipulation of Notch signaling in experimental models. For example, genetic and pharmacological inhibition of Notch signaling was reported to ameliorate pathology in several disease models including, rheumatoid arthritis (RA) [14], atherosclerosis [15], systemic lupus erythematosus (SLE) [16], systemic sclerosis [17], experimental autoimmune encephalomyelitis [18], and infectious diseases [19,20,21].

Despite the numerous reports that support a crucial role for Notch signaling in many inflammatory disorders, the mechanistic role of Notch signaling in these conditions requires more elucidation. However, significant progress has been reported in the involvement of Notch signaling in immunity through regulation of immune cells development and function [3]. Table 1 summarizes the key findings from studies that investigated the role of Notch signaling in lymphocytes development, maturation, differentiation, and activation.

## 2. Notch Signaling in Macrophages

Although the role of Notch signaling in lymphocytes development, maturation, differentiation, and activation has been comprehensively reported, less is known about the role of Notch signaling in the development, differentiation, and activation of myeloid cells including macrophages [3,7]. For a long time, macrophages were thought to be sender cells that only express Notch ligands and activate the Notch signaling in heterotypic-receiver cells. However, significant findings were published recently that advanced these understandings. For example, several studies have reported the expression of Notch receptors 1–4 and ligands in human- and murine-derived primary macrophages [32,33,34]. The expression of several proteins involved in Notch post-transitional modulation, processing, and signaling, including Kuzbanian, Fringe, and Presenilin has been reported in macrophages [32]. This was confirmed by immunohistochemical and ultrastructural studies, which revealed direct membrane contact between neighboring macrophages in human atherosclerosis plaques [35]. Moreover, the upregulation of DLL-4 and multiple Notch signaling components have been reported within macrophages in human atherosclerosis plaques [36]. In addition to the heterotypic role of DLL-4 in activated macrophages [37], Fung et al. demonstrated a homotypic role of DLL-4 in macrophages through the four Notch receptors ligation [36]. Clearly, these data support the notion that Notch signaling mediate homotypic and heterotypic juxtacrine communication in macrophages because of macrophage-macrophage and macrophage-stromal cell interaction, respectively. Consequently, macrophages can act as a Notch signal inducer and/or receiver cells to respond to the external microenvironmental cues and signals.

Macrophages are key effector cells of innate immunity that plays an essential role in host defense and inflammatory response to endogenous and exogenous stimuli [38,39]. In addition to their immune functions, macrophages offer a wide array of tissue supportive functions that maintain and regulate development, homeostasis, and tissue repair [38,40,41]. As professional phagocytic cells, macrophages express a wide array of specialized receptors, which recognize and react to potential antigens [42]. For example, macrophages express germline-encoded pattern recognition receptors (PRRs), which recognize conserved molecular-specialized antigens known as pathogen-associated molecular patterns (PAMPs) or host-derived damaged-associated molecular patterns (DAMPs) [41,43]. PRRs include the transmembrane receptors such as TLRs and C-type lectin receptor (CLRs), and cytoplasmic receptors such as nucleotide-binding oligomerization domains (NOD)-like receptors (NLRs) and retinoic acid-inducible gene-I (RIG-I)-like receptors (RLRs) [39,41,44]. PRRs may contribute to pathogen phagocytosis, phagolysosome maturation and they expedite the presentation of antigen or antigenic determinants to other immune cells [39]. Activated PRRs initiate a series of signaling events that ultimately induce the immune system to response to the invading stimuli [39,45]. Specifically, lipopolysaccharide (LPS) in gram-negative bacteria binds to TLR-4, which in turn mediates expression of pro-inflammatory mediators through NF-κB and microtubule-associated protein (MAP) kinase signaling. The mediators through autocrine and paracrine processes activate downstream signaling pathways such as Janus kinase/signal transducers and activators of transcription (JAK/STAT) pathway, which ultimately induce more modulation of the immune response [46]. Dysregulation in PRRs signaling system has been linked to increasing the susceptibility to various bacterial and viral infections [47,48] and the development of inflammatory and autoimmune diseases. This includes rheumatic diseases [49], asthma [50], and inflammatory bowel disease (IBD) [44,51].

### 2.1. Macrophage Polarization

Depending on the local environmental cues, macrophages can presume a variety of structural and functional phenotypes, which allow them to reveal a spectrum of functional specialism throughout the body [38,52]. Mirroring the Th1/Th2 activation of T cells, macrophages may undergo either classical (M1) or alternative (M2) activation based on variable activation signals. M1 inflammatory macrophages are characterized by expression of a high level of pro-inflammatory cytokines and toxic reactive oxygen intermediates, such as tumor necrosis factor (TNF)-α, IL-6, monocyte chemoattractant protein (MCP)-1, and inducible nitric oxide synthase (iNOS) [53,54,55]. M1 macrophages demonstrate combating functions against a variety of bacteria, viruses, and protozoa, and involve in anti-tumor immunity [38]. In contrast, M2 macrophages display immunoregulatory and pro-tumor properties and activated by distinct cues including IL-4 and IL-13 [53]. M2 macrophages have a significant role in wound repair and healing, parasite Infection control, and the resolution of inflammation through the production of anti-inflammatory cytokines IL-10 and IL-1RA and enhance the production of Arginase-1 enzyme, which is important in ameliorating the detrimental nitric oxide production [38,54,56].

The disparity of macrophages activation is linked to several pathological disorders including cancer [57], infection [58,59], and autoimmunity [60]. For example, Excessive M1 macrophage polarization may induce atherogenesis, insulin resistance, and adipose inflammation [15]. This is due to the ability of M1 macrophage to induce systemic inflammatory state by upregulation IL-6, MCP-1, and TNF-α, which altogether impaired insulin sensitivity and magnify the mechanisms that favor atherosclerotic plaque formation and development [61,62]. Additionally, impaired M1/M2 balance in the mucosa of Crohn’s disease (CD) patients diminish enterocyte differentiation and impair mucosal regeneration [63]. In rheumatic diseases, the M1 phenotype is prevalently seen in RA, osteoarthritis, Behcet’s disease, and gout associated with dramatic pro-inflammatory cytokines production. On the other hand, M2 macrophages are more prevalent in spondyloarthritis and systemic sclerosis, associated with tissue remodeling and angiogenesis [64]. Increased M1/M2 macrophage ratio was reported in patients with severe influenza infection correlated with disease severity among those patients [65]. Understanding the signaling pathways that control macrophages differentiation, activation, metabolism, proliferation, and apoptosis is important for explaining the molecular basis of many pathological conditions and designing novel macrophage-mediated therapeutic approaches.

### 2.2. Notch Signaling in Macrophages during Inflammation and Infection

The role of Notch signaling in macrophages during inflammation and infection is well reported [36,66,67,68]. Notch signaling has been shown to favor the inflammatory microenvironment and steer the macrophages pro-inflammatory responses in different inflammatory settings [36,69]. For example, hypoxia-inducible factor (HIF)-1α, a transcription factor upregulated in hypoxia and inflammatory microenvironments, was reported to induce the expression of Notch ligands (DLL-4 and Jagged-1) in macrophages [63]. This study also reported a positive correlation between M1/M2 ratio and Notch signaling in the mucosa of chronic CD patients [63]. Bai et al. reported the upregulation of Notch-1 signaling in the LPS-induced septic mice model [66]. They also reported that myeloid-specific Notch-1 knockout caused diminish the upregulation of pro-inflammatory mediators, which resulted in ameliorates organ damage and dysfunction in septic mice [66]. Furthermore, DLL-4 was reported to mediate macrophages and endothelium interaction during microvascular inflammation [37]. This is consistent with published data that activated Notch signaling is detected in macrophages within atherosclerosis plaque [36], inflamed synovium [69,70], and diabetic wounds [71]. On the other hand, the association of Notch signaling in monocytes/macrophages with bacterial and viral infections was pinpointed by many reports. For instance, the upregulation of DLL-1 and Notch target genes in primary human monocytes was reported following in vitro gram-positive and gram-negative bacterial infection. Furthermore, the induction of Notch signaling was reported following infection of macrophages with an intracellular bacillus like *Mycobacterium bovis* bacillus Calmette-Guérin (BCG) [21] and injection of macrophages with tuberculin purified protein derivative (PPD) [72].

Notch signaling seems to negatively regulate the immune response and defense mechanisms against mycobacterial infection. Notch inhibitor decreases bacteria burden and pathological complications in the lungs mice infected with *M. tuberculosis* [73]. On the other hand, Ito et al. reported the implication of Notch signaling in the influenza H1N1 virus infection. Specifically, they reported the upregulation of DLL-1 in macrophages during H1N1 infection, which mediated an anti-viral effect by regulating the IFN-γ expression from CD4^+^ and CD8^+^ T cells [67].

Despite of all these advancements, which highlighted the critical role of Notch signaling in macrophages during inflammation and infection, the mechanism(s) by which the bidirectional interaction of Notch signaling in macrophages and inflammation and infection remained unclear. 

### 2.3. Reciprocal Modulation of Notch Signaling and TLRs-Signaling

Macrophages express a variety of PRRs including TLRs that allow them to recognize and respond to the invading pathogens and direct the innate and adaptive immune responses [41,43]. Notch receptors and ligands are constitutively expressed in macrophages [32,33,34], hinting the critical role of Notch signaling in those cells. As both pathways are associated with inflammation and infection, instant activation, and reciprocal modulation of both signaling pathways seem acceptable. In terms of TLR-mediated modulation of Notch signaling, TLRs might modulate Notch signaling indirectly through inducing the expression of Notch receptors and ligands, which in turn activate the Notch signaling pathway. Many studies reported the enhancement of Notch receptors and ligands expression in response to TLRs activation [32,74,75]. Palaga et al. reported the upregulation of Notch-1 signal in BCG-infected and tuberculin purified protein derivative (PPD)-treated macrophages via TLR-2-MyD88 axis-dependent manner [72,75]. Mycobacterial infection through TLR-9 induces the expression of DLL-4 during pulmonary granuloma formation as reported by Ito.et al [68]. Interestingly, in granulomatous lungs, *Tlr9^−/−^* mice the mRNA expression of IL-10 was enhanced while TNF-α was decreased coincidence with decreased DLL-4 expression [68]. Foldi et al. reported on the interaction between Notch and TLRs pathways in inducing the expression of Jagged-1 in primary human and mouse macrophages, which was mediated by RBP-J and NF-κB in human macrophages and RBP-J and MAPKs signals in mouse macrophages [74]. In contrast to Jagged-1, DLL-4 has been induced in TLR-stimulated macrophages in RBP-J independent manner [74]. Hu et al. reported direct TLRs-mediated induction of the canonical Notch target genes, HES-1 and HEY-1 in primary human macrophages, through histone phosphorylation at the site of Notch target gene loci [76]. The bidirectional modulation between Notch and TLRs-mediated signaling was also reported by several studies. For example, Monsalve et al., reported a positive feedback loop between Notch and TLR signaling and discovered that Notch-1 upregulation was observed in LPS-induced murine macrophages [77]. They also reported that active NICD enhanced basal and LPS-induced NF-κB activation through increased NF-κB inhibitor degradation and enhanced NF-κB nucleolus translocation and DNA binding [77]. Notch signaling is inhibited constitutively by γ-secretase inhibitor *N*-[*N*-(3,5-Difluorophenylacetyl-l-alanyl)]-*S*-phenylglycine t-Butyl ester (DAPT) attenuated NF-κB activity in LPS-induced macrophages [77]. These findings were supported by many studies that collectively agree on the ability of Notch signaling to augment TLR-associated inflammatory responses in vitro and in vivo and diminish this inflammatory response following Notch signaling inactivation [36,75,76,78]. Contrary to these reports, Zhang et al. reported that Notch signaling negatively regulates TLR-associated inflammatory responses in mouse peritoneal macrophages. They reported that overexpression of NICD-1 and NICD-2 suppressed TLR-4 mediated pro-inflammatory cytokines production such as TNF-α and IL-6 and promoted anti-inflammatory cytokines IL-10. This was due to reducing ERK phosphorylation, which negatively affects NF-κB transcriptional activity [79].

### 2.4. Reciprocal Modulation of Notch Signaling and Cytokines

**Figure 2:**
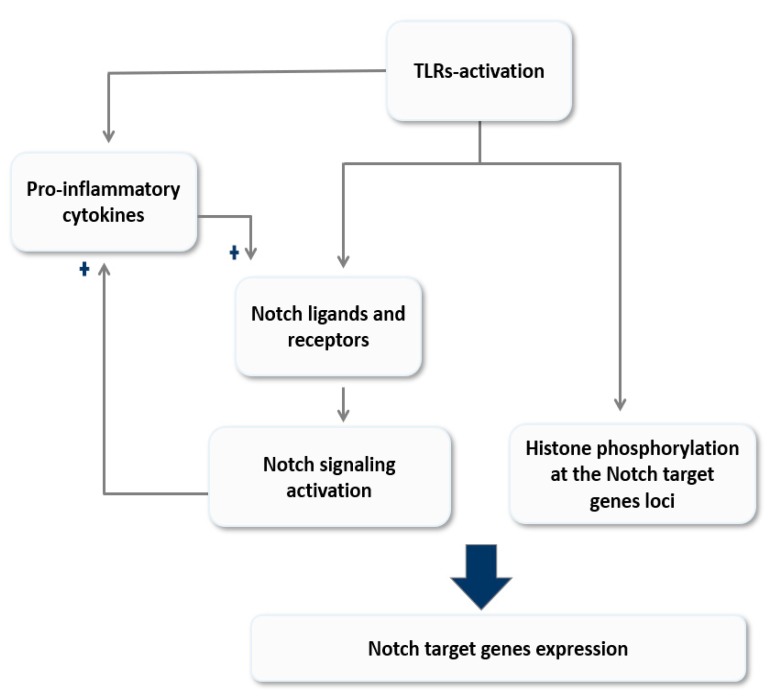
A model of reciprocal modulation of Notch signaling and Toll-like receptor (TLR)-signaling in macrophages. Activation of TLRs modulates Notch signaling either directly through histone modification at the site of Notch target genes loci and/or indirectly by inducing the expression of its ligands and receptors. On the other hand, Notch signaling magnifies the TLR-mediate inflammatory responses by enhancing the expression of pro-inflammatory cytokines; these cytokines in turn amplify the feed-forward loop by inducing Notch ligands expression. This model explores the role of Notch signaling in sustain TLR-mediated responses and its implication in inflammation chronicity.

Cytokines are potent signaling molecules that primarily function to orchestrate local and systemic immune response during health and disease [80]. However, dysregulation of cytokines production and signaling can mediate harmful and pathogenic effects. Interestingly, many studies reported a reciprocal relationship between cytokines and Notch signaling in macrophages, which further emphasized the critical role of Notch signaling in regulating macrophages immune response. Fung et al. reported increase expression of DLL-4 in response to pro-inflammatory cytokines IL-1β but not TNF-α and INF-γ in human primary macrophages [36]. On the other hand, INF-γ, the most effective activator of macrophages, robustly enhances the expression of Jagged-1, and decreases DLL-1 and DLL-4 expression in primary human and mouse macrophages [74]. Another study reported that INF-γ could induce Notch-1 and not Jagged-1 [32]. In the H1N1 virus infection model, the autocrine INFα-receptor activation mediates STAT1/2-induced transcription of DLL-1 in macrophages [67]. Consistently, in gram-positive and gram-negative bacterial infection models, IL-6 was noted to induce the expression of DLL-1 in primary human monocytes through activation of transcription factor STAT-3. In turn, DLL-1 was shown to enhance IL-6 production and subsequently activate STAT-3 [46]. Of course, Notch signaling seems to directly regulate IL-6 expression. For example, chromatin immunoprecipitation (Chip) assay revealed the association of Notch-1 with IL-6 promoter in LPS/INF-γ induced macrophages [81]. Over-expression of NICD-1 in mouse macrophages has also increased the expression of the pro-inflammatory cytokines TNF-α and IL-6 [77]. Moreover, Notch signaling induced IL-12 expression directly by c-Rel and indirectly by TNF-α production [82]. It is well known that repression of Notch signaling has negatively regulated various pro-inflammatory cytokines, such as TNF-α, IL-1β, and IL-6 [36,75,76,78]. As shown in Figure 2, these studies imply a positive feed-forward loop between Notch signaling and various inflammatory stimuli in macrophages. TLRs activation either directly and/or indirectly induces Notch signaling activation, which in turn, amplifies TLR-mediated pro-inflammatory responses through inducing expression of pro-inflammatory cytokines such as TNF-α and IL-6. These cytokines then positively feed this loop by inducing the expression of Notch ligands. Collectively, this amplification loop could sustain the inflammatory response in macrophages for further time point, which strongly explained the implication and involvement of Notch signaling in many chronic inflammatory conditions.

### 2.5. Notch Signal in Macrophages Activation and Functions

Emerging evidence indicates the contribution of Notch signaling in M1 macrophage polarization, leading to overexpression of TNF-α, iNOS, IL-6, and MCP-1 [15,77]. Using Chip assay, direct interaction between NICD and TNF-α, IL-6 and iNOS promoters was observed [77,81]. Other studies reported that pharmacologically and genetically inhibition of Notch signaling led to the suppression of M1 macrophages polarization and activation of M2 macrophages and subsequent anti-inflammatory cytokines production [75,77,83,84]. Interestingly, inhibition of Notch signaling resulted in M2 macrophage polarization even in the presence of M1 inducers [83]. On the other hand, DLL-4, a marker for M1 macrophages, impeded IL-4 induced M2 polarization through inhibition of M2 genes expression and selective induction of cells apoptosis during M2 polarization [85]. It was reported that Notch receptors (Notch1-3) and ligands (DLL-1, DLL-4, and Jagged-1) were upregulated in M1 macrophages compared to M2 and undifferentiated macrophages [86].

**Figure 3:**
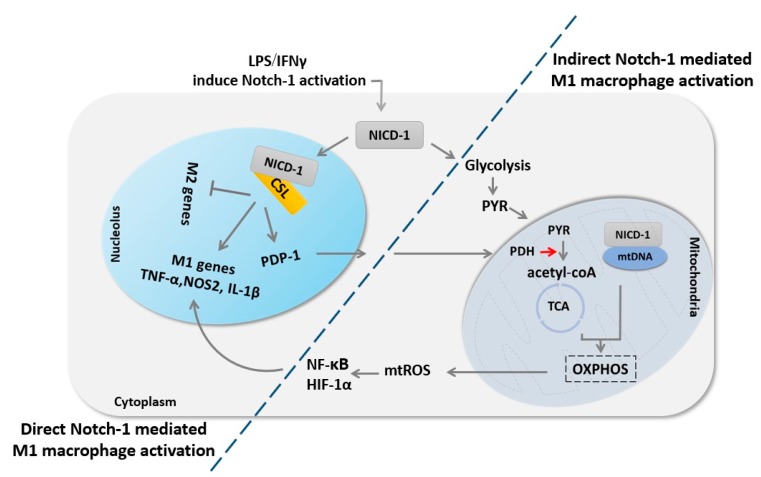
Notch-1 signaling regulates macrophage M1 fate through direct transcriptional regulation and indirect metabolic regulation. First, Notch-1 signaling activation results in the release of NICD-1, which translocates into the nucleus to induce M1 macrophage genes expression. Concurrently, Notch-1 activation enhances glycolysis combined with enhancing PDP-1 expression and subsequent PDH activity, which ultimately increases glucose flux to the tricarboxylic acid (TCA) cycle. NICD-1 also translocates to the mitochondria and induces mitochondria DNA (mtDNA) expression, glucose oxidation, mitochondrial oxidative phosphorylation (OXPHOS), and consequent mitochondrial reactive oxygen species (mtROS), which in turn induce NF-κB and HIF-1α activation, which results in augmentation of M1 macrophage genes expression.

Despite the overwhelming data that support the pivotal role of Notch signaling in macrophage polarization, the mechanisms by which Notch signaling implicating its effect still far to be elucidated. Xu et al., clearly demonstrated that Notch-RBP-J signaling promotes M1 macrophage polarization by directly regulated the expression of transcription factor interferon regulatory factor 8 (IRF8) [87]. Besides, other studies reported that Notch signaling could indirectly promote M1 macrophage polarization by integration with other signaling pathways such as NF-κB and MAPK [75,76]. Interestingly, macrophage metabolic analysis revealed that Notch-1 signaling promotes M1 polarization through reprogramming macrophage metabolism to glycolysis. Xu et al. coupled transcriptional activation of M1 genes with upregulation of mitochondrial oxidative phosphorylation (OXPHOS) and reactive oxygen species (mtROS). Whereas enhanced recruitment of NICD-1 to nuclear and mitochondrial genes induce the expression of pyruvate dehydrogenase (PDH) phosphatase-1 (PDP)-1and electron transport chain components (NADH dehydrogenase, cytochrome b, cytochrome c oxidase, and ATP synthases), which altogether enhances glucose oxidation, OXPHOS, and consequent mtROS which in turn activate HIF-1α and NF-κB to induce M1 macrophage activation [88] (Figure 3).

## 3. Notch Signaling in RA Pathogenesis

RA is an autoimmune inflammatory joint disorder, characterized by macrophages and lymphocytes infiltration, synoviocyte hyperplasia, and progressive joint destruction [89]. The role of Notch signaling in the pathogenesis of RA has been reported. Genome-wide association studies have identified rs874040^CC^ locus in the *RBP-J* gene, a key canonical Notch signaling mediator as one of RA susceptibility loci [90]. Furthermore, enhanced expression and activation of Notch signaling components have been detected in synovial tissue [91,92], vascular endothelial cells [93], and peripheral lymphocytes [94] in patients with RA. Notch signaling has been shown to be implicated in various RA pathogenesis processes. For instance, it has been reported to mediate TNFα-induced RA synoviocytes proliferation [91], and accelerate production of pro-inflammatory cytokines and immune responses including upregulation of anti-type II collagen (CII) antibodies [14]. Additionally, Notch signaling has been noted to mediate vascular endothelial growth factor (VEGF)/angiopoietin-2 (Ang-2) and hypoxia-induced angiogenesis and invasion in inflamed RA joint [93,95]. Moreover, Notch-3 and DLL-1 have been known to mediate CII-specific T-cells expansion and alter its response, which is usually elevated during the early phase of RA pathogenesis [96]. On the other hand, genetic and pharmacological inhibition of Notch signaling demonstrated relief in RA severity and had reduced pro-inflammatory cytokines levels in RA synoviocytes and collagen-induced arthritis (CIA) mice [93,97,98]. Interestingly, joints directed nanoparticles that bear either pharmacological or genetic Notch inhibitors successfully attenuate the severity of RA by reducing the progression of inflammation, and delay bone and cartilage damage in CIA mice [99,100]. These studies suggest that Notch signaling plays an essential role in RA pathogenesis. However, it remained unclear what specific cell type may be governed by Notch signaling in inflamed RA joints.

Macrophages play a pivotal role in RA pathogenesis, evident by the numerous numbers and clear activation state of macrophages in synovial tissue, which are significantly correlated with disease severity [101,102]. Macrophages exhibit extensive pro-inflammatory, destructive, and remodeling properties, which significantly contribute to acute and chronic stages of RA pathogenesis [102,103]. Despite the ample evidence supporting the contribution of macrophages in RA pathogenesis, there is a lack of knowledge about the macrophage subsets in the RA synovial tissue. However, pro-inflammatory cytokines such as TNF-a and IL-1, which are consistently produced by M1 macrophages, are expressed abundantly in RA, whereas M2 characteristic cytokines such as IL-10 and IL-4 are relatively diminished in patients with RA [104]. Interestingly, M1 macrophages were predominately observed in high disease activity RA patients, whereas M2 macrophages are associated with low disease activity or clinical remission RA [105]. Most recently, the imbalance between M1 and M2 macrophages is considered one of the main causes of RA [106,107]. On the other hand, targeting unbalanced macrophage polarization may hold promise for treating RA by re-establishing homeostatic macrophages equilibrium. For example, the administration of human umbilical cord blood stem cells ameliorated the severity of CIA by promoting M2 macrophage polarization and suppresses the activation of M1 macrophages [108]. Besides, alginate nanoparticles loaded with IL-10 plasmid DNA and specifically designed to target macrophages have efficiently reduced the progression of inflammation and joints damage in experimental arthritis by re-polarizing macrophages from M1 to M2 phenotype [109]. Interestingly, many effective RA medications have been reported to manipulate M1/M2 polarization in favor of M2 macrophage polarization [110]. Consequently, cutting down M1 macrophage and promoting M2 macrophage polarization could offer a favorable treatment paradigm for RA.

**Figure 4:**
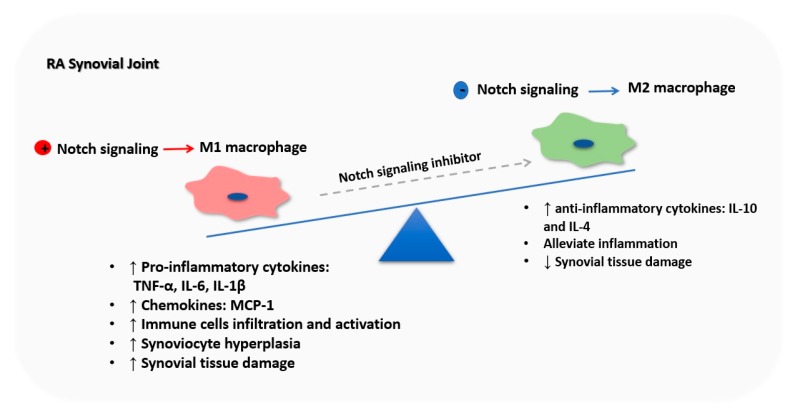
Notch signaling orchestrates macrophage polarization in rheumatoid arthritis (RA) synovial joint. RA synovium microenvironment induces Notch activation in BM-derived macrophages, inducing their M1 polarization. M1 macrophages are the major producer of pro-inflammatory cytokines such as TNF-α, IL-6, and IL-1β and chemokines such as MCP-1 driving immune cells infiltration and activation, synoviocyte proliferation, tissue destruction and bone loss in the RA synovial joint, which exacerbates RA symptoms. Nevertheless, inhibition of Notch signaling promotes M2 polarization, decreasing M1 macrophages number, and releasing anti-inflammatory cytokines, leading to RA symptoms.

Given the implication of Notch signaling in the pathogenesis of RA and the crucial role of Notch signaling in the polarization of the macrophages, Notch signaling seems to play a causal role in M1/M2 imbalance in RA, which significantly implies in RA pathogenesis. Using (TNF-α)-transgenic/(Hes-1)-GFP mice as RA model bearing Notch reporter transgene, Sun et al. identified M1 macrophages derived from bone marrow (BM) as the main cells with activated Notch signaling in the inflamed joint of (TNF-α)-transgenic mice. Additionally, they reported that RA synovial tissue promotes the activation of Notch signaling in BM-derived macrophages, leading to M1 polarization. While thapsigargin (Notch inhibitor) reduces TNF-α induced M1 macrophage polarization and attenuates inflammation and joint bone loss by switching M1 to M2 macrophages [70] (Figure 4). In the same context, low expression of microRNA (miR)-146a (the Notch-1 inhibitor [111]) was reported in the LY6C^high^ monocyte subset of CIA mice, associated with increased arthritis severity and bone erosion. Whereas, in vivo delivery of miR146-a mimic to LY6C^high^ monocyte rescue the bone erosion in CIA mice [112]. Interestingly, miR-146a promotes M2 macrophage polarization and diminish M1 macrophage polarization by targeting Notch-1 signaling in RAW264.7 macrophages [84]. This clearly suggests a pivotal role for Notch signaling in myeloid lineage in RA pathogenesis. Overall, targeting Notch signaling in myeloid lineage may represent a potential novel therapeutic target for RA by controlling the balance of M1 and M2 macrophage polarization and re-establishing the homeostatic immune milieu. However, many clinical and pre-clinical studies are warranted to establish their therapeutic amenability in RA. 

## 4. Potentials and Challenges in Manipulating Notch Signaling for Therapeutic Applications

Numerous evidence supports the involvement of Notch signaling in the pathogenesis of many inflammatory conditions [9,10,11,12,13]. In most instances, targeting Notch signaling has been shown to ameliorate inflammation and minimize associated tissue damage [14,15,66]. Therefore, targeting Notch signaling could be a powerful and promising strategy to combat many inflammatory conditions. The involvement of several enzymatic steps in the activation and regulation of Notch signaling offers many potential therapeutic targets to manipulate Notch signaling for therapeutic purposes. This includes inhibition of receptor cleavage by γ-secretase inhibitors and blockage of Notch receptors or ligands with specific antibodies. Importantly, the crosstalk between Notch signaling and other signaling pathways offers opportunities for combinatorial treatment to target many pathways simultaneously, which may augment the therapeutic benefits [113]. Despite these potentials, more research is needed to overcome the many challenges for using Notch signaling as therapeutic targets. For example, targeting Notch signaling by γ-secretase inhibitor has been shown to produce off-target effects resulting in toxicity and inadequate drug efficacy [114]. Limited knowledge about the functions and expression status of Notch ligands and receptors in different pathological conditions add additional hurdles to use them for therapeutic applications. Thus, further studies are needed to elucidate the precise functions of Notch receptors and ligands and their biological relevance. New strategies are needed, which include discoveries of new targets, novel antagonists, and most importantly unique delivery approaches. For example, RA nanoparticle system used for targeting Notch signaling by DAPT at the site of inflammation demonstrated high therapeutic efficacy compared with DAPT systemic administration [99]. This clearly confirm that specific targeting approaches against Notch signaling at the site of inflammation by nanoparticle technology could offer a promising therapeutic approach for many intractable diseases. 

## 5. Conclusions

Notch signaling is essential in all cellular processes, and its dysregulation has been linked to many pathological disorders including RA. A growing body of literature confirmed the involvement of Notch signaling in immunity through regulation of immune cells development and function. Although much remains unclear about the role of Notch signaling in macrophages, numerous lines of evidence support the important role of Notch signaling in macrophages inflammatory response to various stimuli. Interestingly, the role of Notch signaling in macrophages is not seemed ON/OFF effect, but it operated in an oscillatory feedback loop to intensify the inflammatory response and define their final outcome. As Notch signaling is easily amenable to pharmacological manipulation, targeting Notch signaling could offer a powerful and promising strategy to ameliorate inflammation in many pathological settings. Targeting Notch signaling has been shown to ameliorate inflammation and minimize associated tissue damage in many pathological conditions, including RA. However, there are still many challenges for using Notch signaling as therapeutic targets. Further studies are needed to elucidate the mechanistic role of various Notch receptors and ligands in various inflammatory conditions, which will provide a better understanding of how Notch signaling drives immune response under these conditions and offer a new therapeutic target to inflammatory disorders such as RA.

## Figures and Tables

**Table 1:** Key findings about the role of Notch signaling in lymphocyte development, differentiation, and activation.

Stage	Ligand	Receptor	Notch Pathway	Effect	Ref.
**Development**	DLL4	Notch1	Canonical	T cell lineage commitment and maturation	[22]
	DLL1	Notch2	Canonical	Marginal zone B cell development	[23]
**Differentiation and Activation**	DLL ligands	Notch3	Non-Canonical	Th1 expansion, Increase T-bet expression and IFN-γ production	[24,25]
	Jagged ligands	Notch1	Canonical	Th2 differentiation, increase IL-4 production	[25]
	Dll4	Nocth3	Canonical	Th17 differentiation, increase RORγt expression and IL-17 production	[26,27]
	Jagged-2, DLL4	Notch3	Canonical	Treg differentiation, Foxp3 upregulation	[28,29]
	DLL1	Notch1, Notch2	Canonical	Cytotoxic T cell differentiation, increase IFN-γ production	[30,31]

T helper type (Th), T-box protein expressed in T cells (T-bet), Interferon-γ (IFN-γ), Interleukin (IL), Retinoic acid-related orphan receptor gamma t (RORγt), Regulatory T cells (Treg), and Forkhead box P3 (Foxp3).
